# Early Development of Locomotor Patterns and Motor Control in Very Young Children at High Risk of Cerebral Palsy, a Longitudinal Case Series

**DOI:** 10.3389/fnhum.2021.659415

**Published:** 2021-06-03

**Authors:** Annike Bekius, Margit M. Bach, Laura A. van de Pol, Jaap Harlaar, Andreas Daffertshofer, Nadia Dominici, Annemieke I. Buizer

**Affiliations:** ^1^Department of Human Movement Sciences, Faculty of Behavioural and Movement Sciences, Amsterdam Movement Sciences & Institute for Brain and Behavior Amsterdam, Vrije Universiteit Amsterdam, Amsterdam, Netherlands; ^2^Department of Rehabilitation Medicine, Amsterdam Movement Sciences, Amsterdam Universitair Medisch Centrum, Vrije Universiteit Amsterdam, Amsterdam, Netherlands; ^3^Department of Pediatric Neurology, Amsterdam Universitair Medisch Centrum, Vrije Universiteit Amsterdam, Amsterdam, Netherlands; ^4^Department of Biomechanical Engineering, Delft University of Technology, Delft, Netherlands; ^5^Emma Children's Hospital, Amsterdam Universitair Medisch Centrum, Vrije Universiteit Amsterdam, Amsterdam, Netherlands

**Keywords:** development of walking, early brain lesions, electromyography, muscle synergies, intersegmental coordination

## Abstract

The first years of life might be critical for encouraging independent walking in children with cerebral palsy (CP). We sought to identify mechanisms that may underlie the impaired development of walking in three young children with early brain lesions, at high risk of CP, via comprehensive instrumented longitudinal assessments of locomotor patterns and muscle activation during walking. We followed three children (P1–P3) with early brain lesions, at high risk of CP, during five consecutive gait analysis sessions covering a period of 1 to 2 years, starting before the onset of independent walking, and including the session during the first independent steps. In the course of the study, P1 did not develop CP, P2 was diagnosed with unilateral and P3 with bilateral CP. We monitored the early development of locomotor patterns over time via spatiotemporal gait parameters, intersegmental coordination (estimated via principal component analysis), electromyography activity, and muscle synergies (determined from 11 bilateral muscles via nonnegative matrix factorization). P1 and P2 started to walk independently at the corrected age of 14 and 22 months, respectively. In both of them, spatiotemporal gait parameters, intersegmental coordination, muscle activation patterns, and muscle synergy structure changed from supported to independent walking, although to a lesser extent when unilateral CP was diagnosed (P2), especially for the most affected leg. The child with bilateral CP (P3) did not develop independent walking, and all the parameters did not change over time. Our exploratory longitudinal study revealed differences in maturation of locomotor patterns between children with divergent developmental trajectories. We succeeded in identifying mechanisms that may underlie impaired walking development in very young children at high risk of CP. When verified in larger sample sizes, our approach may be considered a means to improve prognosis and to pinpoint possible targets for early intervention.

## Introduction

Cerebral palsy (CP) is a neurodevelopmental disorder caused by non-progressive brain lesions before birth or in the first year of life (Himmelmann and Uvebrant, 2018). The distribution of CP can be unilateral or bilateral, depending on the site of the brain lesion. CP covers a wide clinical spectrum of mobility levels, varying from walking independently to being completely wheelchair dependent. Functional mobility in CP is classified using the Gross Motor Function Classification System (GMFCS) (Palisano et al., 1997). The levels of the GMFCS range from I to V, with children at level I and II ultimately walking without aids and children at level III needing a walking aid. Children at levels IV and V (primarily) use a wheelchair for their mobility.

Walking is the most important mode of locomotion in everyday life. Typically developing (TD) children take their first independent steps (FS) between the age of 9 and 18 months, but this milestone is often delayed or not achieved in children with CP. Developmental stages of locomotion usually occur at a later age in children with CP compared to TD children (Largo et al., 1985; Meyns et al., 2012). The maturation of walking patterns is reflected in both kinematic and neuromuscular measures (Dewolf et al., 2020). Understanding the mechanisms that underlie abnormal development of independent walking (IW) is important for the design of early interventions that aim at improving function mobility in children with CP.

Older children with CP appear to retain some of the characteristics of the younger TD children during the early phases of the development of walking (Berger et al., 1984; Leonard et al., 1991). In TD children, the foot trajectory during swing (the time course of the vertical foot displacements) develops starting from a prominent centered single-peak foot lift in toddlers to the stereotyped double-peaked trajectory with a minimum foot clearance during midswing in older children and healthy adults (Dominici et al., 2007). Indeed, the mature two-peaked foot trajectory reflects the accurate endpoint control strategy that is the result of the intersegmental coordination in both limbs (Winter, 1992; Ivanenko et al., 2002). By contrast, in children with CP, a single-peak foot lift similar to TD toddlers may persist, often specific to the most affected side in unilateral CP (Cappellini et al., 2016). While in TD children the intersegmental coordination of the lower limb segments quickly develops (Cheron et al., 2001; Ivanenko et al., 2004a, 2007; Dominici et al., 2011)—typically, intersegmental coordination develops rapidly in the first few months after the onset of IW (Cheron et al., 2001)—in children with CP, this may be less pronounced (Leonard et al., 1991; Berger, 1998). Apparently, intersegmental coordination matures less or much slower in children with CP. In unilateral CP, this has been shown to be specific to the most affected body side (Cappellini et al., 2016).

One way to assess neuromuscular control during walking in healthy individuals and patient populations is through muscle synergy analysis. Walking requires refined neuromuscular coordination, and the central nervous system arguably simplifies neuromuscular control during walking by the recruitment of groups of muscles, called muscle synergies or locomotor modules (Ivanenko et al., 2005; Hart and Giszter, 2010; Dominici et al., 2011; Bizzi and Cheung, 2013). During typical development, the number of basic activation patterns increases from two, during neonate stepping, to four, when children walk independently (Dominici et al., 2011; Sylos-Labini et al., 2020). Muscle activity patterns during walking in older children with CP seem to match the patterns of TD toddlers (Cappellini et al., 2016), suggesting that also the maturation of the activation of individual muscles lags behind in children with CP. School-age children with CP recruit fewer muscle synergies compared to TD children, and it seems that they employ “simpler” neuromuscular control strategies (Steele et al., 2015; Tang et al., 2015; Schwartz et al., 2016; Shuman et al., 2016, 2017, 2018, 2019; Goudriaan et al., 2018; Hashiguchi et al., 2018; Bekius et al., 2020). In children with CP, the temporal structure of muscle synergies, i.e., their activation patterns, largely agrees with that of TD toddlers; i.e., they contain wider activation bursts compared to older TD children.

Combining kinematic and neural measures can provide additional insight into motor development and may help to detect early motor deficits (Dewolf et al., 2020). Yet, previous studies investigating locomotor patterns in young children with CP had a cross-sectional design (Cappellini et al., 2016, 2018), which may limit inferences about how changes in neuromuscular control are related to the altered development of walking. In addition, a limited number of recorded muscles in previous studies (Steele et al., 2013; Tang et al., 2015; Shuman et al., 2016, 2017; Bekius et al., 2020) may limit the conclusions on the spatiotemporal structure of muscle activity patterns. A more detailed and comprehensive assessment of multimuscle coordinated patterns is needed (Damiano, 2015). The aim of the current exploratory longitudinal study was to investigate the development of locomotor patterns and motor control during walking in very young children with early brain lesions, at high risk of CP. The focus was on development starting before the onset of IW, covering a period of 1 to 2 years, with the aim to record the emergence of the FS. We studied locomotor patterns using kinematic analysis and motor control using muscle synergy analysis. We hypothesized (1) intersegmental dependency and muscle synergy structure to change over time after IW onset and (2) the development of gait kinematics and muscle synergies to be delayed in children with a diagnosis of CP. We also expected (3) differential changes of gait kinematics on the most vs. the least affected side when unilateral CP was diagnosed, and (4) the number of synergies to be reduced the more a child was affected by CP.

## Materials and Methods

### Participants

Three children with early brain lesions, at high risk of CP and not yet walking independently, were included in this longitudinal study. The participants were recruited from the Department of Pediatric Rehabilitation and the Department of Pediatric Neurology at the Amsterdam University Medical Centers (Amsterdam UMC, location VUmc). The study was approved by the Medical Ethics Committee of the Amsterdam UMC (location VUmc) (NL59589.029.16). The parents of all children were informed about the procedure of the study and provided written informed consent prior to participation in accordance with the declaration of Helsinki for medical research involving human participants.

### Study Design

Experiments were performed in the clinical gait laboratory of the Department of Rehabilitation Medicine at the Amsterdam UMC, location VUmc. The laboratory setting and experimental procedures were adapted to the children, such that the risks were equal or lower to that of walking at home. The responsible investigators, one or both parents of the child, and a pediatric physiotherapist were present during the experiments.

Every participant underwent five consecutive gait analysis sessions within a period of 1–2 years, to record development from supported walking (SW), to FS, to IW. SW sessions were conducted prior to IW onset. There, the pediatric physiotherapist, experimenter, or parent supported the child during stepping attempts by its trunk or arms. FS sessions were recorded when the children performed their first unsupported steps. They were scheduled within 2 weeks of first unaided walking experience, as reported by the parents. All sessions recorded after the FS session were labeled IW (Table 1).

**Table 1:** Participant characteristics.

**Participant**	**Gender**	**Session**	**Walking ability**	**Age (mo)**	**CA (mo)**	**WA (mo)**	**Distribution**	**Subtype**	**GMFCS**	**Brain damage**	**BW (kg)**	**BL (cm)**	**N strides**
P1	F	1	SW	12.7	12.7	−1.8	Bi	Spastic	HR	Infection	7.8	75	37
		2	FS	15.2	15.2	0.8	Bi	Spastic	HR		8.8	74	47
		3	IW	17.5	17.5	3.1	Bi	Spastic	I		9.4	80	62
		4	IW	22.3	22.3	7.9	Bi	Spastic	I		10.6	85	42
		5	IW	27.2	27.2	12.8	Bi	Undef	n/a		11.0	87	33
P2	M	1	SW	14.5	14.6	−6.6	Uni R	Spastic	HR	Media infarction	11.1	84	0
		2	SW	19.2	19.3	−1.4	Uni R	Spastic	HR		12.0	84	52
		3	FS	22.1	22.2	0.3	Uni R	Spastic	I		12.4	91	29
		4	IW	24.6	24.7	2.8	Uni R	Spastic	I		12.0	89	40
		5	IW	26.9	27	5.1	Uni R	Spastic	I		12.4	90	54
P3	F	1	SW	31.7	30.0	—	Bi	Spastic	III	PVL	12.7	85	60
		2	SW	35.4	33.7	—	Bi	Spastic	III		12.8	90	36
		3	SW	42.8	41.1	—	Bi	Spastic	III		14.0	95	59
		4	SW	49.1	47.4	—	Bi	Spastic	III		15.0	105	48
		5	SW	56.1	53.6	—	Bi	Spastic	III		12.6	105	51

*F, female; M, male; SW, supported walking; FS, first steps; IW, independent walking; mo, months; CA, corrected age; WA, walking age (expressed as time since onset of independent walking); Bi, bilateral; Uni, unilateral; R, right; undef, undefined; GMFCS, gross motor function classification system; n/a, not applicable; HR, high risk; asym, asymmetric; sym, symmetric; PVL, periventricular leukomalacia; BW, body weight; BL, body length; N, number*.

All children walked barefoot on a treadmill during SW sessions and over ground during FS and IW sessions. If children wore ankle-foot orthoses in daily life, these were removed during the experiment. The treadmill speed was adjusted to induce a walking pattern and turned to a comfortable speed for the child. During FS and IW sessions, children were encouraged to walk in a straight line at a comfortable walking speed.

### Data Acquisition

**Figure 1:**
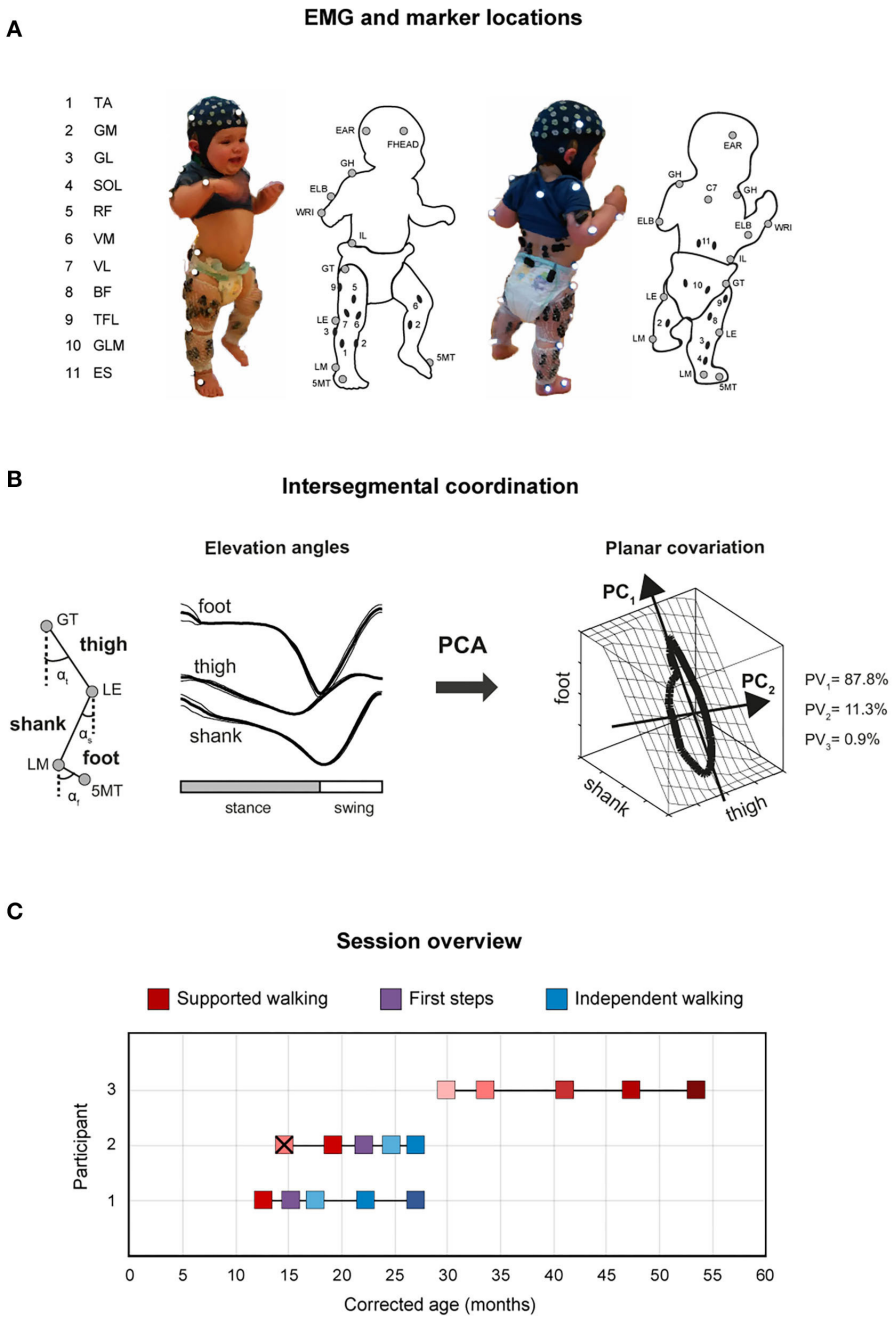
Experimental setup and gait analysis session overview. **(A)** Marker locations are denoted by gray circles, and EMG electrode locations by black ovals. 5MT, head of fifth metatarsophalangeal joint; LM, lateral malleolus; LE, lateral femur epicondyle; GT, greater trochanter; IL, iliac crest; WRI, ulnar styloid; ELB, lateral humeral epicondyle; GH, glenohumeral joint; C7, seventh cervical vertebra; EAR, ear; FHEAD, forehead; TA, tibialis anterior; GM, gastrocnemius medialis; GL, gastrocnemius lateralis; SOL, soleus; RF, rectus femoris; VM, vastus medialis; VL, vastus lateralis; BF, biceps femoris; TFL, tensor fascia latae; GLM, gluteus maximus; ES, erector spinae at L2 level. **(B)** Schematic illustration of principal component (PC) analysis of elevation angles of lower limb segments (thigh: α_*t*_, shank: α_*s*_, foot: α_*f*_), and its corresponding gait loop plotted three-dimensionally in one representative typically developing child (4 years old) during walking. A loop is obtained by plotting the thigh elevation angle vs. shank and foot elevation angles (after mean values subtraction). The shape and orientation of the gait loop reflect the coordination of limb segments as described by PC_1_ (reflects the largest variance, which corresponds to the length of the gait loop), and PC_2_ (the second largest variance, which corresponds to the width of the loop), here indicated with black arrows. Percentage of total variation explained by the three PCs (PV_1_-PV_3_) are indicated. Note in adult and older children walking, two principal components typically account for ~99% of the total variance (PV_3_ = 0.9% in the example). **(C)** Gait analyses over time (corrected age, in months) for P1–P3 showing supported walking (SW: shades of red), first steps (FS: purple), and independent walking (IW: shades of blue). Different color shades from light to dark indicate the change in age and/or independent walking experience. P3 did not start to walk independently. X means the child did not take any steps during that session.

During every session, full-body kinematics were recorded bilaterally and sampled at 100 Hz using a VICON system (Oxford, UK) with 12 cameras surrounding the 10-m-long walking area. Reflective markers (diameter 14 mm) were attached to the skin of the participants representative of anatomical reference points overlying the following bony landmarks on both sides of the body (Figure 1A): glenohumeral joint (GH), seventh cervical vertebra (C7), ear (EAR), forehead (FHEAD), ulnar styloid (WRI), lateral humeral epicondyle (ELB), iliac spinal crest (IL), greater trochanter (GT), lateral femur epicondyle (LE), lateral malleolus (LM), and head of fifth metatarsophalangeal joint (5MT). In addition, videos were recorded at 50 Hz using the VICON system using two cameras placed in the sagittal plane.

Muscle activity was assessed by means of surface electromyography (EMG) from 11 pairs of bilateral leg and trunk muscles, i.e., tibialis anterior (TA), gastrocnemius medialis (GM), gastrocnemius lateralis (GL), soleus (SOL), rectus femoris (RF), vastus medialis (VM), vastus lateralis (VL), biceps femoris (BF), tensor fascia latae (TFL), gluteus maximus (GLM), and erector spinae at L2 level (ES). Mini-golden reusable surface EMG disc–electrode pairs (15-mm-diameter electrodes, acquisition area of 4 mm^2^) were placed at the approximate location of the muscle belly on the cleaned skin, with interelectrode spacing of ~1.5 cm. EMG electrode placement was performed according to the Surface Electromyography for the Non-Invasive Assessment of Muscles protocol (Hermens et al., 2000) and the recommendations for minimizing cross-talk between adjacent muscles that were described in detail previously (Ivanenko et al., 2004b, 2005; Dominici et al., 2011). Preamplified EMG sensor units were attached to the skin and fixed with elastic gauzes, to minimize movement artifacts (Figure 1A). Two Cometa Mini Wave wireless 16-channel EMG systems (Cometa, s.r.l, Italy) were used, and EMG signals were sampled at 1 kHz. Electroencephalography (EEG) recordings were made but not analyzed here. Sampling of kinematic, video, and EMG signals was synchronized.

### Data Analysis

The gait patterns were described by (1) kinematics: (a) spatiotemporal gait parameters, including walking velocity, stride duration, and double support duration; (b) intersegmental coordination, estimated via principal component analysis (PCA) of the elevation angles of thigh, shank, and foot segments (Borghese et al., 1996); and (2) neuromuscular control: EMG activity and muscle synergies characteristics, described by the full width at half maximum (FWHM) of the muscle activity, temporal activation patterns of the muscle synergies, and variability accounted for (VAF). Gait initiation and termination, as well as jumps and turns, were discarded from analysis. The lower body was modeled as an interconnected chain of rigid segments: GT-LE for the thigh, LE-LM for the shank, and LM-5MT for the foot. All analyses were conducted in MATLAB (version 2020a, MathWorks Inc., Natick, MA, USA).

#### Spatiotemporal Gait Parameters

Step events were manually defined based on the video recordings and later confirmed using the kinematic data. We defined a stride from foot strike to foot strike of the ipsilateral foot, stance duration as the percentage of the gait cycle from foot strike to foot off. Stride velocity was computed using the corresponding stride length (three-dimensional displacement of the LM marker) and duration of both legs. Data were time-interpolated over individual gait cycles to fit a normalized 201-point time base.

#### Intersegmental Coordination

We constructed elevation angles of the thigh, shank, and foot segments (α_*t*_, α_*s*_, and α_*f*_, respectively) to correspond with angles between the segment projected on the sagittal plane and the vertical, i.e., to be positive in the forward direction when distal markers fell anterior to the proximal one. In healthy adults and older children, these elevation angles describe a path (gait loop) that lies close to a plane that is defined by their eigenvectors (Figure 1B). To examine the development of intersegmental coordination (the gait loop and the corresponding plane) across sessions within subjects, PCA was applied to lower limb elevation angles of each gait cycle, for each session and participant separately (Borghese et al., 1996; Bianchi et al., 1998; Ivanenko et al., 2007; Dominici et al., 2010). Briefly, we computed the covariance matrix of the thigh–shank–foot elevation angels (after subtraction of the mean value of each angular coordinate) across the gait cycle. The three principal components (PC_1_-PC_3_) including the explained variance (PV_1_-PV_3_) and eigenvectors (**u**_**1**_–**u**_**3**_), were obtained (Figure 1B). In general, the first two eigenvectors lie on the best-fitting plane of angular covariation, with **u**_**2**_ orthogonal to **u**_**1**_, whereas the third eigenvector, **u**_**3**_, is the normal to the covariation plane and defines the plane orientation. For every eigenvector, the parameters u_it_, u_is_, and u_if_ correspond to the direction cosines with the positive semiaxis of the thigh, shank, and foot angular coordinates, respectively. The percentages of variance accounted for by the second (PV_2_) and third (PV_3_) eigenvectors were assessed and compared between sessions, as well as the rotation of the normal to the plane by using the u_3t_ parameter (Bianchi et al., 1998; Dominici et al., 2007, 2010; Cappellini et al., 2018).

#### Muscle Activity

EMG signals were notch and high-pass filtered (fourth-order Butterworth 50 ± 0.01 Hz, and >30 Hz, respectively), full-wave rectified, and subsequently low-pass filtered (fourth-order Butterworth <10 Hz) to obtain linear envelopes. For some sessions, EMG data from few muscles had to be discarded because of sustained artifacts after filtering. Possible contamination of the EMG recordings could be detected by the electrical cross-talk due to volume conduction of activity across adjacent muscles. This issue is particularly relevant when recording many muscles in young children because of their small body size and the resulting close spacing of nearby muscles. Nevertheless, the small size of the EMG electrodes used in our settings and the chosen interelectrode distance should have minimized the pickup from adjacent muscles. Anyway, we attempt to quantify the potential electrical cross-talk by performing a cross-correlation analysis of selected pairs of adjacent flexor/extensor muscles. Cross-correlation was computed after notch (50 Hz) and high-pass (30 Hz) filtering the EMG data to remove any possible power line noise and movement artifacts. We identified the peak of the normalized cross-correlation >0.3 between flexors and extensors as suspect cross-talk (d'Avella et al., 2011; Dominici et al., 2011) in 0.1% to 9.9% of strides, although the values of the correlation coefficients were generally not very high (Supplementary Table 3). We verified that the removal of those strides potentially affected by cross-talk did not change any conclusion drawn from those analyses.

The data were time-normalized for each limb to *t* = 201 data points per gait cycle. The FWHM of the muscle activity was determined based on the minimum-subtracted envelopes of muscle activity patterns (Alves-Pinto et al., 2016; Cappellini et al., 2016, 2018; Bach et al., 2021), separately for every muscle, gait cycle, and session. To ease comparison of our experimental finding with literature, we also reported the FWHM of the mean muscle activity for each muscle and session (Supplementary Material 1). In case of muscle activations with more than one peak, the peak with the largest maximum was chosen as the main peak. The FWHM quantifies the duration of the burst of activity as it equals the sum of the durations of the intervals in which the EMG signal exceeded half of their maximum. That is, the larger the FWHM, the longer the muscle contraction is sustained.

#### Muscle Synergies

Per session, EMG amplitudes of every muscle were normalized to the maximum of their mean value across strides plus its standard deviation (SD). Non-negative matrix factorization (NMF) was applied to the unilateral gait-cycle averaged EMG envelopes to extract muscle synergies. NMF decomposes the EMG into *i* = 1, …, *n* temporal activation patterns *H*_*i*_ and corresponding synergy weights *W*_*i*_ by mean of the linear combination

$$\begin{array}{l} EMG=\overset{n}{\underset{i=1}{\sum }}H_{i}W_{i}+e, \end{array}$$

where the preprocessed *EMG* data (*m* × *t* matrix, where *m* is the number of muscles, here 11, and *t* is the number of time points, here 201) are a linear combination of the temporal activation patterns *H* (*n* × *t* matrix, where *n* < *m* is a predetermined number of synergies, see below) and synergy weights *W* (*m* × *n* matrix), and *e* denotes the residual error.

The reconstruction accuracy of the extracted synergies was determined by calculating the percent of VAF, which is the ratio of the sum of squared errors to the total sum of squares computed with respect to the mean (Dominici et al., 2011; Cappellini et al., 2016). In order to compare the synergy patterns between sessions, we aimed to fix the number of synergies per participant across sessions, by varying the number of synergies from 1 to 8 and exploring the VAF slopes and synergy patterns across sessions. In addition, we investigated VAF by one synergy (VAF1, Steele et al., 2015; Shuman et al., 2019) and compared this parameter between sessions.

To quantitatively characterize differences in the duration of synergy activation patterns, we also computed the corresponding FWHM (Cappellini et al., 2016, 2018; Bach et al., 2021), in line with the approach of the muscle activation patterns (see above).

### Statistical Analysis

Statistical analyses were performed using SPSS (IBM SPSS Statistics for Mac, version 26.0; IBM Corp., Armonk, NY, USA). All data are reported as mean (±SD). Normality was assessed using Q–Q plots, and homogeneity of variance was tested using a Levene test. Development of spatiotemporal gait parameters, intersegmental coordination values, and FWHM per muscle across sessions within each participant was assessed using a linear mixed-effects model (LMM, Molenberghs and Verbeke, 2000). LMM corrects for interdependency of repeated measures within one participant using random effects, and the number of observations per sessions can vary. We fitted an LMM with PV_2_, PV_3_, u_3t_, stance duration, double support, velocity, and FWHM per muscle as outcome variables. *Session* was included as fixed and *trial* as random effect. To test whether development of the outcome variables differed between sides in P2, the interaction between session and side was included for P2. For all analyses, *p* < 0.05 was considered statistically significant.

## Results

### Medical History

Child P1 (female) was born at term after an uneventful pregnancy. Birth was complicated by umbilical cord entanglement and perinatal asphyxia with Apgar scores of 4 and 5, respectively, after 1 and 5 min, respectively. Clinically, she recovered quickly, and there was no indication for therapeutic hypothermia. Because of a possible infection, she was treated with antibiotics for 7 days, but blood cultures remained negative. Twenty-two hours after birth, she developed severe neonatal convulsions, for which the child was treated with phenobarbitone, midazolam, lidocaine, and levetiracetam. A magnetic resonance imaging (MRI) scan at the age of 4 days revealed extensive bilateral signal changes in the white matter of the frontal, temporal, parietal, and occipital lobes; corticospinal tracts; internal capsule; and thalamus. A probable diagnosis of hypoxic–ischemic encephalopathy due to perinatal asphyxia was made. Neurological examination at the age of 8 months showed a mild developmental delay and a mild paresis of the left arm. At follow-up, until the age of 3.5 years, development appeared normal, and no further abnormalities were found via neurological examination, excluding a diagnosis of CP.

Child P2 (male) was also born at term after an uneventful pregnancy. The child developed normally in the first 3 months of life, although it was noted that the right arm appeared stiffer when parents changed clothes. When 3 months old, asymmetry in upper extremity motor function was established. At the age of 5 months, he developed West syndrome for which he was successfully treated with levetiracetam and vigabatrin. Neurological examination at the age of 8 months showed spastic paresis of the right arm with hyperreflexia of the knee tendon reflex at the right side. An MRI scan of the brain at the age of 8 months showed a congenital infarction of the medial cerebral artery at the left side. A diagnosis of unilateral spastic CP, GMFCS I, was made.

Child P3 (female) was born preterm after 33 weeks and 1 day of pregnancy. At the age of 3 weeks, she suffered a group B streptococcal infection. She showed a developmental delay and rolled over for the first time at the corrected age of 11 months, and the tone of the lower extremities had been high since the first weeks. An MRI scan of the brain at the corrected age of 12 months showed periventricular leukomalacia, related to prematurity. A diagnosis of bilateral spastic CP, GMFCS III, was made.

### Gait Analysis Session Overview

For P1 and P2, the sessions covered a period of 14.5 and 12.4 months in between the first and the last session and included one and two SW sessions, respectively, an FS session, and three and two IW sessions, respectively. For P3, the sessions covered a period of 24 months and included five SW sessions (Figure 1C). P1 started to walk at a typical age, i.e., 14 months old, whereas P2 took its FS around the corrected age of 22 months old. P3 did not walk independently yet at the corrected age of 53 months. During the first session of P2 (corrected age 14.6 months), the child did not take any steps; thus, for this participant, only four sessions are reported in the following.

### Spatiotemporal Gait Parameters

Video analysis revealed that P1 had a flat foot strike in sessions 1 and 2 (SW and FS, respectively), whereas both feet showed a heel strike from session 3 (IW) onward. P2 had a flat foot strike of both feet from sessions 2 to 4 (SW, FS, and IW, respectively), and during session 5 (IW), only the least affected leg showed a heel strike. P3 had a toe landing and was crossing her legs in all sessions.

**Figure 2:**
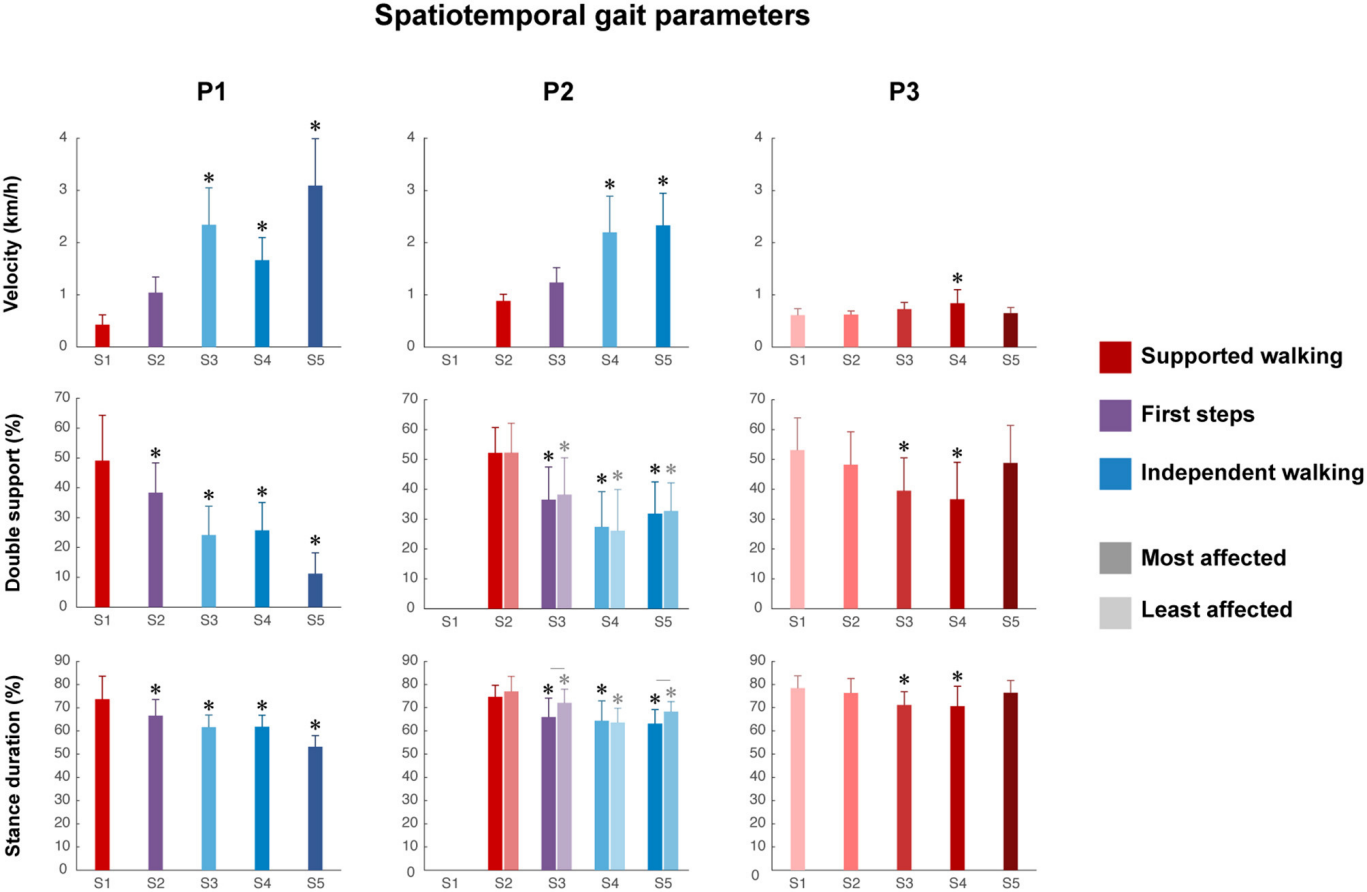
Spatiotemporal gait parameters. Velocity, percentage of double support and stance duration from S1 to S5. Error bars indicate SDs. For the percentage of double support and stance duration of P2, the most affected side (dark color) and least affected side (light color) are shown, whereas for P1 and P3, one side is shown. Significant linear differences across sessions are denoted by asterisks, and significant interactions between session and side are denoted by lines between the most and least affected sides for P2 (*p* < 0.05).

Walking velocity showed a significant main effect of session in all children (P1, *p* < 0.001; P2, *p* < 0.001; P3, *p* < 0.01). For P1, a significant increase was found from SW session 1 to IW sessions 3 to 5 (Figure 2 and Supplementary Table 1). For P2, a significant increase was found from SW session 2 to IW sessions 4 to 5. For P3, a significant increase was found between SW sessions 1 and 4, but walking velocity decreased again in session 5.

The percentage of double support phase showed a significant main effect of session in all children (P1, *p* < 0.001; P2, *p* < 0.01; P3, *p* < 0.001). For P1, a significant decrease was found between SW session 1 and all sessions (Figure 2 and Supplementary Table 1). For P2, a significant decrease was found between SW session 2 and all sessions, in both the most and least affected side. For P3, a significant decrease was found between SW session 1 and SW sessions 3 to 4, but the percentage of double support phase increased again in session 5.

The percentage of relative stance duration showed a significant main effect of session in all children (P1, *p* < 0.001; most affected side P2, *p* < 0.05; least affected side P2, *p* < 0.001; P3, *p* < 0.001). For P1, a significant decrease was found between SW session 1 and all sessions (Figure 2 and Supplementary Table 1). For P2, a significant decrease was found between SW session 2 and all sessions in both the most affected and least affected side, although the developmental slope differed between sides, as shown by a significant interaction effect between session and side (*p* < 0.01). For P3, a significant decrease was between SW session 1 and SW sessions 3–4, but the percentage of relative stance duration increased again in session 5.

### Intersegmental Coordination

**Figure 3:**
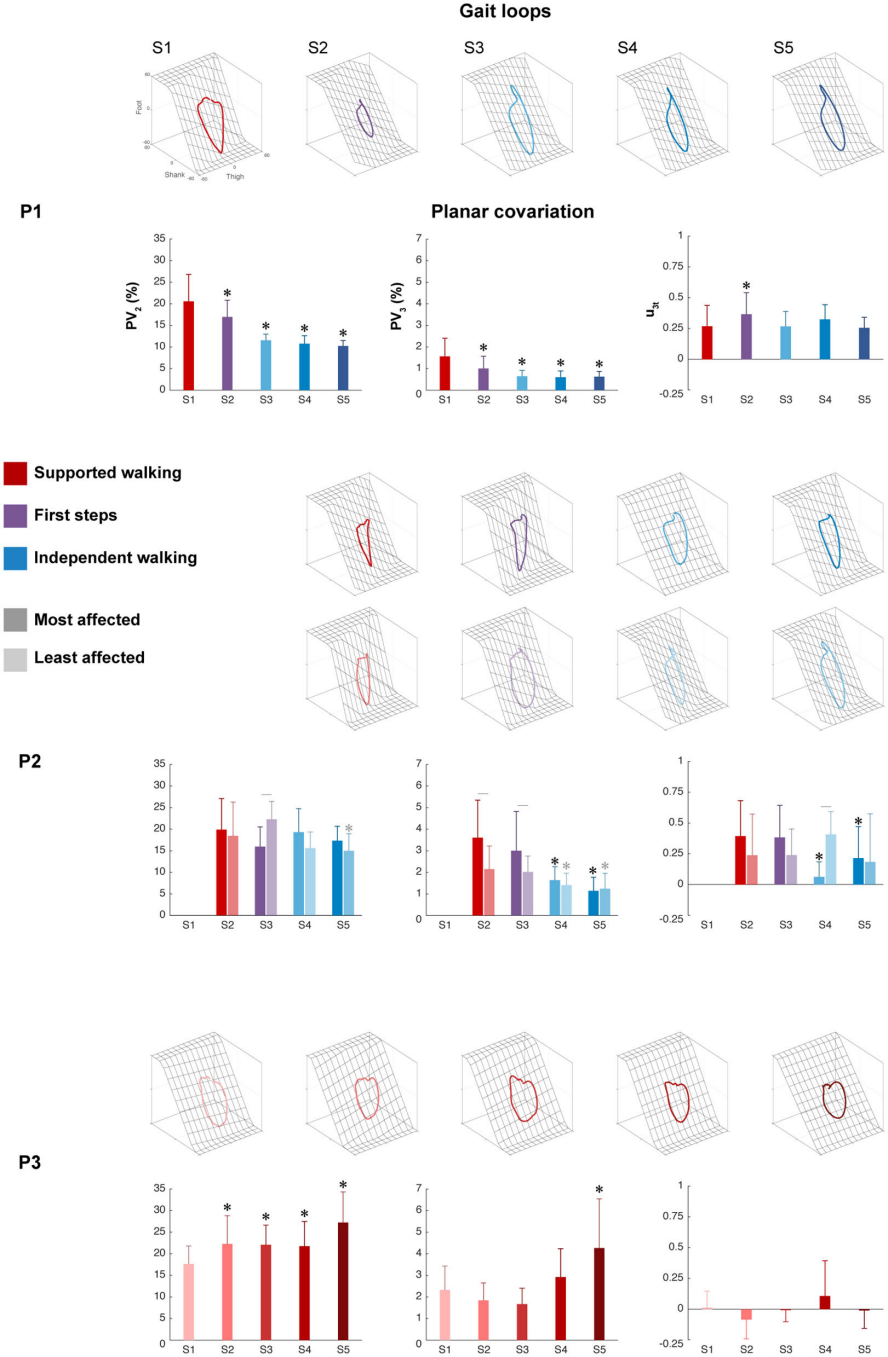
Intersegmental coordination. Gait loops plus the corresponding mean percentage of total variance explained by the second and third eigenvectors (PV_2_ and PV_3_), and the mean orientations of the normal to the plane (u_3t_) are shown from S1 to S5 for each participant. Error bars indicate SDs across strides. For P2, the most affected side (dark color) and the least affected sides (light color) are shown, whereas for P1 and P3, one side is shown. Significant differences with session 1 are marked by asterisks, and significant interactions between session and side are denoted by lines between the most and least affected sides for P2 (*p* < 0.05).

The intersegmental coordination of the thigh–shank–foot elevation angles was compared across sessions for the three participants and was evaluated using PCA. The planar covariation of the leg elevation angles is directly related to the dimensionality of the original data set, and the method is shown in Figure 1B. In summary, Figure 3 shows the mean gait loops for all sessions of each participant and its corresponding values of planar covariation.

The percentage of variance accounted for by the second eigenvector (PV_2_) showed a significant main effect of session in P1 (*p* < 0.001), the least affected side of P2 (*p* < 0.01), and P3 (*p* < 0.001), but not in the most affected side of P2 (*p* = 0.110). For P1, a significant decrease was found between SW session 1 and all sessions, indicating a reduction in the width of the gait loop from SW to IW, which is also visible in the changes of gait loops for the IW sessions compared to the SW session (Figure 3 and Supplementary Table 1). The least affected side of P2 showed a significant decrease between SW session 2 and IW session 5, but an increase during FS session 3. In contrast, P3 showed a significant increase between SW session 1 and all sessions, but the gait loops showed similar shapes in all the sessions.

The percentage of variance accounted for by the third eigenvector (PV_3_), which quantified the planarity of the gait loop, showed a significant main effect of session in P1 (*p* < 0.001), the most and least affected sides of P2 (*p* < 0.001 and *p* < 0.01, resp.) and P3 (*p* < 0.01). For P1, a significant decrease was found between SW session 1 and all sessions, indicating a reduction in the deviation from planarity (Figure 3 and Supplementary Table 1). For P2, a significant decrease was found between SW session 2 and IW sessions 4 to 5 in both the most and least affected side. In contrast, P3 showed a significant increase in PV_3_ between SW sessions 1 and 5.

The orientation of the covariance plane (u_3t_) showed a significant main effect of session in P1 (*p* < 0.001), the most and least affected sides of P2 (*p* < 0.001 and *p* < 0.01, resp.) and P3 (*p* < 0.01). For P1, a significant increase was found between SW session 1 and FS session 2, whereas the most affected side of P2 showed a significant decrease between SW session 1 and IW sessions 4 to 5 (Figure 3 and Supplementary Table 1). In both the least affected side of P2 and in P3, there was no significant difference between session 1 and the other sessions. The analysis showed a significant session interaction effect between session and side for PV_2_, PV_3_, and u_3t_ (*p* < 0.001 for all) in P2, indicating that these parameters developed differently over time for the most and least affected side.

### Muscle Activity

**Figure 4:**
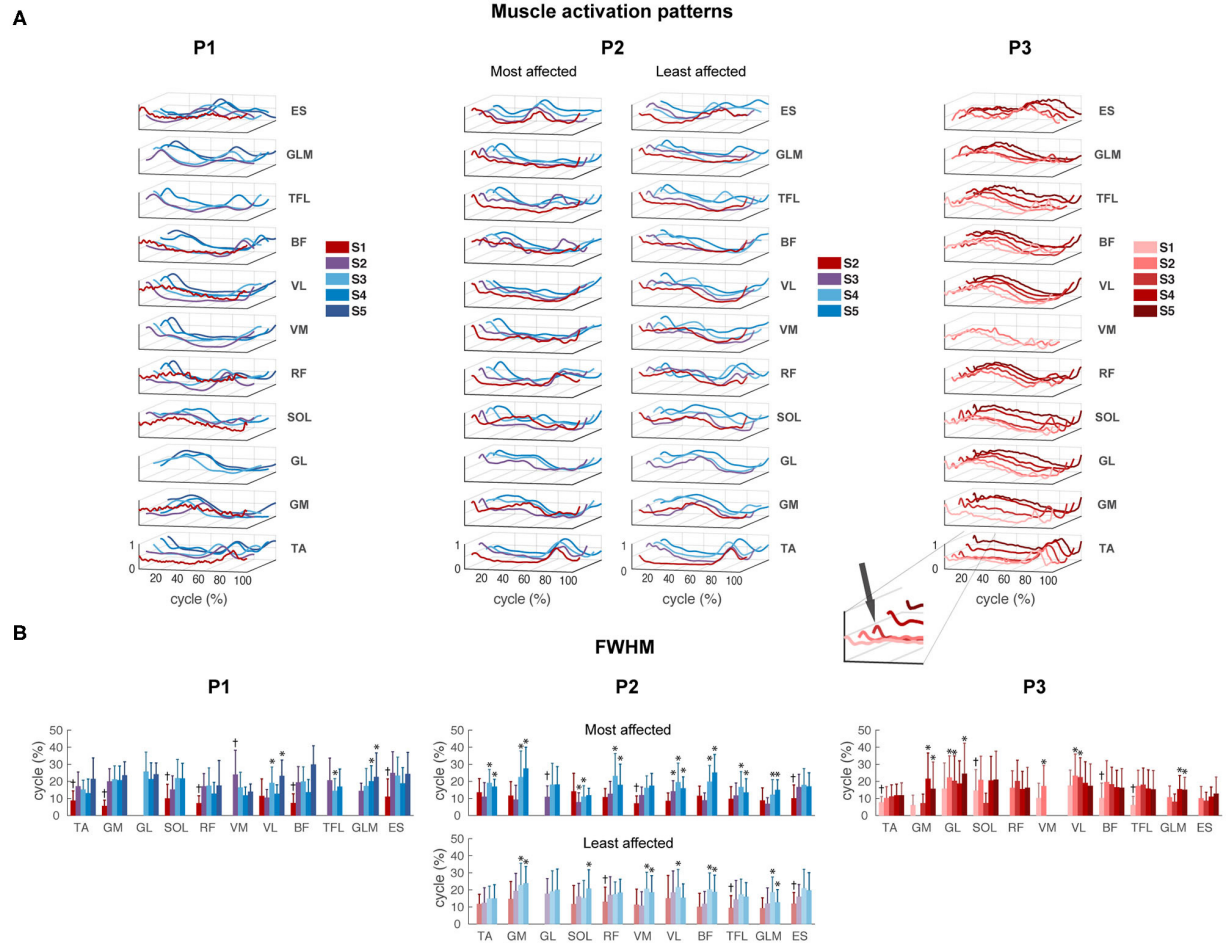
EMG activity. **(A)** Muscle activation patterns averaged across strides per session for P1–P3, for the most and least affected legs in P2. The peak around foot strike is emphasized in the zoom-in view of the TA muscle of P3. **(B)** Full-width of the half maximum (FWHM) per muscle per session in P1–P3. TA, tibialis anterior; GM, gastrocnemius medialis; GL, gastrocnemius lateralis; SOL, soleus; RF, rectus femoris; VM, vastus medialis; VL, vastus lateralis; BF, biceps femoris; TFL, tensor fascia latae; GLM, gluteus maximus; ES, erector spinae at L2 level. *Significant difference compared to the first recorded session of this muscle. ^†^All sessions are significantly different from the first recorded session.

Despite the large variability in EMG activity between strides per muscle, we could observe a clear modification between different sessions in P1 and P2. All activation patterns became smoother and displayed increasingly distinct peaks (Figure 4A). A short burst of activity was present around foot strike in the TA and calf muscles (GM, GL, and SOL) of P3 during all sessions (see zoom-in view of P3 Figure 4A), in the SW and FS sessions of P2, in particular in the most affected leg. In P1, this activity was absent.

For P1, FWHM showed a significant main effect of session for all muscles except GL (*p* = 0.072). FWHM of TA, GM, SOL, RF, VL, BF, and ES significantly increased from SW session 1 to IW sessions 3 to 5, and of GLM from FS session 2 to IW sessions 4 to 5. In contrast, FWHM significantly decreased from FS session 2 to IW session 3 for TFL, and to sessions 3 to 4 for VM (Figure 4B and Supplementary Table 2).

For P2, FWHM showed a significant main effect of session for all muscles in the most affected side, and for all muscles except TA (*p* = 0.061) and GL (*p* = 0.682) in the least affected side. In the most affected side, FWHM of TA, GM, GL, RF, BF, and TFL was significantly higher during IW sessions 4 to 5 compared to SW session 2, whereas FWHM of SOL, VM, VL, and ES already increased during FS session 3. In the least affected side of P2, FWHM of GM, VM, BF, and GLM significantly increased during IW sessions 4 to 5 compared to SW session 2, whereas FWHM of RF, TFL, and ES already increased during FS session 3. FWHM of SOL was only significantly larger during IW session 5 compared to SW session 1 (Figure 4B and Supplementary Table 2). An interaction effect between session and side was found for GM, SOL, RF, BF, and GLM (*p* < 0.01), indicating that FWHM of these muscles developed differently in the most compared to the least affected side.

For P3, FWHM showed a significant main effect of session for all muscles (*p* < 0.05), except RF (*p* = 0.120). FWHM of TA, SOL, VM, BF, and TFL was significantly smaller in session 1 compared to all other sessions. FWHM of GM and GLM was significantly larger during sessions 4 to 5 and of GL during sessions 2, 3, and 5, compared to the first recorded session (Figure 4B and Supplementary Table 2). In all participants, variability in FWHM between strides was large.

The results of the FWHM computed on the average muscle activity can be found in Supplementary Material 1. Similar to the results shown in Figure 4B, this analysis revealed clear changes between different sessions in P1 and P2. In contrast, P3 showed similar duration of the main bursts of the mean activity of almost all the muscles between sessions, and on average wider EMG bursts with respect to P1 and P2.

### Muscle Synergies

**Figure 5:**
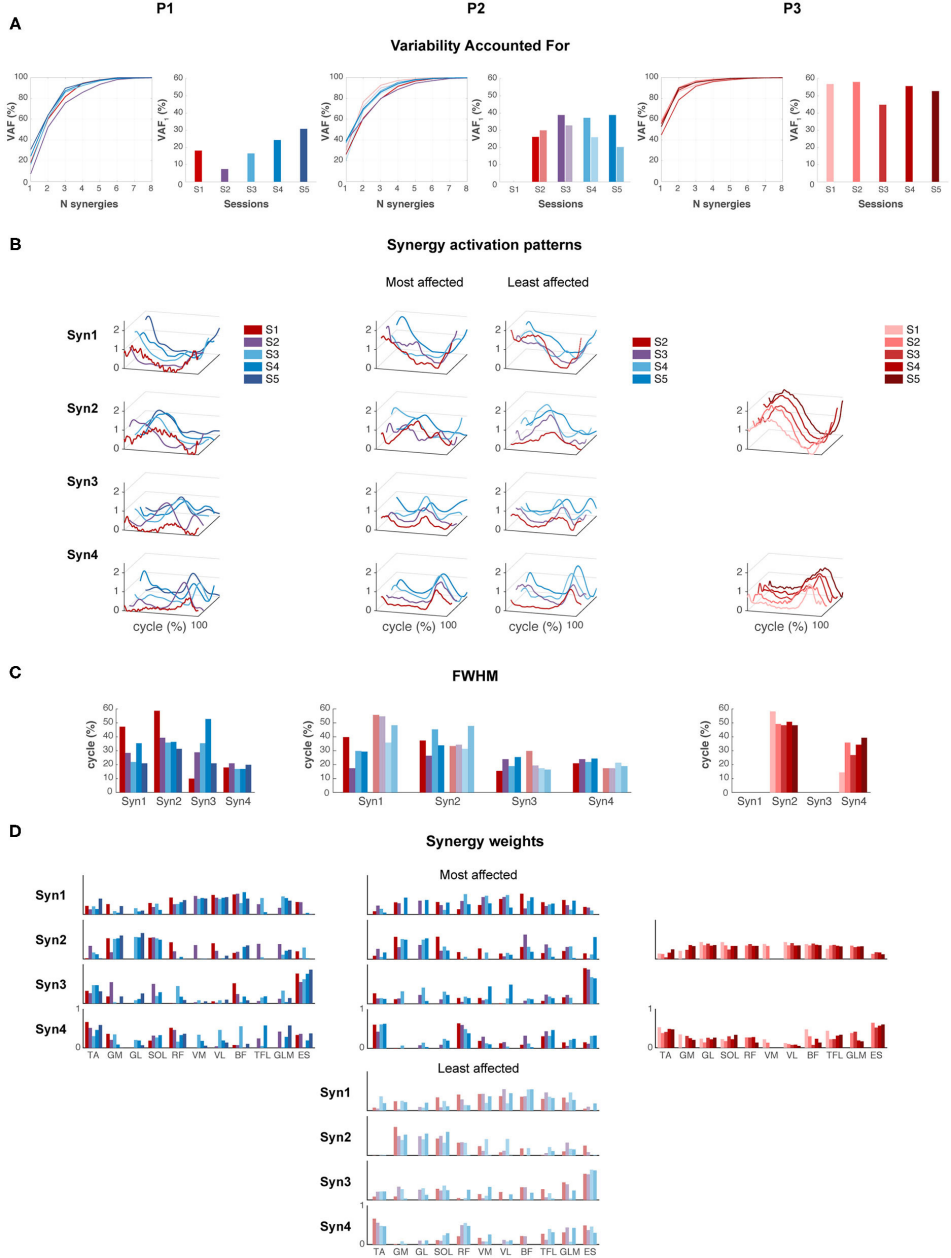
Muscle synergies. **(A)** Variability accounted for (VAF) by one to eight synergies, and for the first synergy (VAF1). **(B)** Synergy activation patterns for a fixed number of synergies: four for P1 and P2, and two for P3. **(C)** Full-width of the half maximum (FWHM) per muscle synergy. **(D)** Synergy weights: contribution of each muscle to each synergy across sessions.

P1 showed an increase in VAF_1_ after FS. The most affected side of P2 showed an increase in VAF_1_ from SW to IW where it stayed constant, whereas the least affected leg showed a decreasing trend in VAF_1_ from FS to IW. VAF_1_ was higher for the most compared to the least affected leg during the FS and IW sessions, but vice versa for the SW session. For P3, VAF_1_ slightly differed between sessions, but there was no clear trend (Figure 5A). Based on the VAF slopes and exploration of the synergy patterns, P1 and P2 generally recruited four synergies across sessions, whereas P3 recruited two synergies. To compare activations patterns across sessions, we fixed the number of synergies of P1 and P2 to four, and of P3 to two.

Synergies were ordered based on the highest correlations between the synergy weights per session (Figures 5B,C). For P1, FWHM of synergy pattern 1 (Syn1) and 2 (Syn2) decreased, i.e., bursts become narrower. By contrast, FWHM of Syn3 increased toward session 4, but decreased again in session 5. Syn4 did not change in FWHM. Despite the changes of synergy patterns of P2, FWHM appeared quite variable between sessions, and we failed to identify any (qualitative) trend. P3 showed an increase in FWHM of Syn4 over time.

Despite the development in synergy activation patterns in P1 and P2, the synergy weights appeared similar across sessions (Figure 5D). In P1 and P2, mainly RF, VM, VL, and BF were active in Syn1, and GM, GL, and SOL in Syn2. ES, and TA to a lesser extent, dominated Syn3, and TA and RF in Syn4. P3 showed simultaneous activations of GM, GL, SOL, RF, VM, VL, TFL, and GLM in Syn2, whereas TA and ES were present in Syn4.

## Discussion

Our longitudinal study captured the early development of walking in three young children at high risk of CP via comprehensive instrumented gait assessments. The exploratory findings illustrate how the combination of kinematic and EMG measures can contribute to our understanding of walking maturation in children with early brain lesions.

We followed three children with divergent development trajectories. Two of them made the transition from SW to IW, whereas the third child had not reached IW at 4.5 years of age. Spatiotemporal gait parameters, intersegmental coordination, and neuromuscular control changed from SW to IW in the child who did not develop CP and the child with unilateral CP. Maturation of gait patterns was delayed in the child with unilateral CP and differed between sides. Walking development appeared entirely absent in the more severely affected child with bilateral CP.

Spatiotemporal gait parameters and intersegmental coordination changed over time from SW to IW in the child without CP, and the child with unilateral CP, although to a lesser extent, whereas there was no development in the child with bilateral CP. This was expected in view of previous research reporting developmental trajectories in children with CP that differ from those in TD children (Berger et al., 1984; Leonard et al., 1991; Berger, 1998). The rapid maturation of planar covariation in the first months after the FS was present whenever children developed IW, irrespective of their age at their first steps. The most affected side of the child with unilateral CP deviated more from planarity than the least affected side during SW and FS, although it was comparable between sides during IW. These results suggest that the intersegmental coordination matured side-specific from just before IW onset during the consecutive 6 months. This period might be devoted to postural gait requirements, whereas in the following years, gait coordination is (merely) refined (Bril and Breniere, 1992; Breniere and Bril, 1998).

In contrast to the rapid maturation of intersegmental coordination after the onset of IW, muscle activity and its major burst duration appeared quite variable between strides in the first 6 to 12 months after the FS. This agrees with previous findings from, e.g., Chang et al. (2006). Probably, toddlers slowly discover how to optimize their muscular activity. In our study, the duration of EMG bursts increased from SW to IW. Cappellini et al. (2016) reported a decrease in FWHM from TD toddlers (1–3 years old) to older TD children, whereas this reduction was not visible in the TD toddler group. The latter falls in the (corrected) age range in our study. Moreover, Cappellini et al. (2016) reported wider EMG bursts in children with CP (2–11 years old), arguably similar to muscle activation patterns in TD toddlers. Similar results were reported by Prosser et al. (2010) in children with CP (2–9 years old) when compared with TD children with similar walking experience (average of 28 months of walking experience). Despite the presence of young children, the age range of the children with CP included in these cross-sectional studies is quite large. In our study, we did not have a TD control group, but we did observe wider average EMG bursts in the more severely affected child with bilateral CP with respect to the children who developed IW. Here we would like to note that gait patterns and their corresponding muscle activity are highly variable in toddlers. It is well possible that a reduction in EMG burst duration occurs later on during development, while children refine their motor pattern (see above). We did notice short bursts of EMG activity immediately after foot strike in the shank and calf muscles of the child with bilateral CP and the calf muscles of mainly the most affected leg of the child with unilateral CP, which might have been caused by spasticity, i.e., hyperreflexive reactions upon muscle stretch following foot strike (Forssberg, 1985).

The control modules that account for muscle activity during walking seemed to develop in the children who developed IW, whereas there were no major changes in the more severely affected child with bilateral CP. The two children walking independently recruited four synergies, whereas the child without the ability to walk independently recruited only two synergies. While gait kinematics improved during IW in the child with unilateral CP, the modulation of groups of muscles to efficiently perform this motor action may have lagged behind. This finds support by previous research reporting the absence of a direct relation between gait kinematics and muscle activations (Buurke et al., 2008) or muscle synergies (Booth et al., 2019). In fact—if true—this implies that gait kinematics and neuromuscular control may follow a different developmental path. The muscle synergy patterns of the child with bilateral CP resemble that of the “primitive” neonate stepping, recruiting two wider synergies with a lot of co-contraction in antagonist muscles (Dominici et al., 2011). Neonate stepping reflects the immature walking pattern that lacks a heel strike and with the tendency to walk on the toes (Forssberg, 1985). Put differently, the child with bilateral CP might still depend mainly on spinal input, whereas supraspinal influence is lacking.

The children were small, especially in the early sessions, yielding limited space for EMG electrodes and, as a consequence, a possible contamination of the EMG recordings due to electrical cross-talk between adjacent muscles that could have affected the data quality. However, the small size of the EMG electrodes used in our experiments and the chosen interelectrode distance should have minimized the pickup from nearby muscles. While it is not possible to dissociate coactivation from cross-talk in adjacent muscles, muscle synergy analysis can identify whether a muscle is activated independent from an adjacent muscle even in the presence of crosstalk. It has been recognized that, if cross-talk did exist, it would likely have affected only the synergy weights and not the number of muscle synergies or the temporal activation patterns (Ivanenko et al., 2004b; Chvatal and Ting, 2013).

In addition, the child with bilateral CP was already 2.5 years old at the time of the first session, whereas it would have certainly been interesting to investigate its walking pattern below that age. Presumably, it would have been a very similar pattern given the little change observed between the ages of 2.5 and 4.5 years for this child.

We identified changes in gait kinematics and neuromuscular control underlying the development of walking in three cases at high risk of CP with divergent developmental trajectories. We showed that such analyses are feasible in very young children. Future longitudinal research with a larger sample of children at high risk of CP could and should provide more insight into the underlying mechanisms of the development of walking. Yet, to establish whether muscle synergies are encoded in the cortex (Zandvoort et al., 2019) or whether they originate solely from spinal cord (Ivanenko et al., 2008; Cappellini et al., 2010) or brainstem (Schepens and Drew, 2004) remains a topic of debate (Bizzi and Cheung, 2013). To solve this puzzle, supplementing our approach by, e.g., synchronous EEG recordings, appears a valid option. Following this idea might be an important step toward the design of early interventions targeting the neural pathways. Combined with the use of novel technologies, such as wearable sensors (Redd et al., 2019; Xu et al., 2019; Airaksinen et al., 2020), and treatments such as feedback training (Booth et al., 2019), and electrical stimulation of muscles, tendons (Sommerfelt et al., 2001; Stackhouse et al., 2007; Wright et al., 2012), or spinal cord (Solopova et al., 2017), during the critical period of walking development, we might become able to improve early identification of motor deficits in children with early brain lesions and identify targets for early intervention to effectively improve walking function in CP.

## Data Availability Statement

The original contributions presented in the study are included in the article/Supplementary Material, further inquiries can be directed to the Corresponding author.

## Ethics Statement

The studies involving human participants were reviewed and approved by Medical Ethics Committee (METc) of the Amsterdam UMC (location VUmc) (NL59589.029.16). Written informed consent to participate in this study was provided by the participants' legal guardian/next of kin.

## Author Contributions

ABe, ABu, and ND designed the study. ABu and LvdP assisted in the recruitment of CP participants. ABe and ND carried out the measurements. ABe, MB, ABu, and ND performed the data analysis, and carried out drafting the manuscript. All the authors have read and concurred with the content in the final manuscript.

## Conflict of Interest

The authors declare that the research was conducted in the absence of any commercial or financial relationships that could be construed as a potential conflict of interest.
