# Supplementary material for: Early Development of Locomotor Patterns and Motor Control in Very Young Children at High Risk of Cerebral Palsy, a Longitudinal Case Series
Source: Front Hum Neurosci. 2021 Jun 3;15:659415. doi: 10.3389/fnhum.2021.659415 (PMC8209291; doi:10.3389/fnhum.2021.659415)
Supplement: Supplementary file 1 [file Table_1.pdf]

Supplementary Table 1. LLM results gait kinematics: estimates of fixed effects session

Participant	Parameter	Session	Estimate (mean±SD)	df	t	p-value	95% CI	
							Lower bound	Upper bound
P1	Velocity	1	.4±0.3	15.9	1.5	-	-0.163	1.012
		2	.6±0.4	15.6	1.6	0.134	-0.212	1.445
		3	1.9±0.3	16	5.9	.000*	1.249	2.641
		4	1.2±0.3	16.1	3.6	.002*	0.5146	1.957
		5	2.6±0.3	16.3	7.7	.000*	1.914	3.361
	DS	1	47.9±2.8	10.2	17.2	-	41.738	54.135
		2	-9.6±3.8	9.3	-2.5	.032*	-18.2	-1.005
		3	-24.0±3.4	11.1	-7.1	.000*	-31.339	-16.581
		4	-22.1±3.5	11.9	-6.3	.000*	-29.813	-14.439
		5	-36.5±3.7	13.8	-10	.000*	-44.338	-28.593
	STdur	1	72.9±1.5	8.9	49.1	-	69.564	76.287
		2	-6.4±2.0	8.1	-3.2	.013*	-11.038	-1.77
		3	-11.4±1.8	10.2	-6.3	.000*	-15.365	-7.359
		4	-11.1±1.9	11.3	-5.8	.000*	-15.286	-6.931
		5	-19.7±2.0	13.7	-9.8	.000*	-23.981	-15.361
	PV_2	1	20.6±0.7	145	28.9	-	19.174	21.985
		2	-3.6±0.9	145	-4.1	.000*	-5.392	-1.869
		3	-9.0±0.8	145	-10.8	.000*	-10.693	-7.376
		4	-9.8±0.9	145	-10.6	.000*	-11.659	-8.005
		5	-10.8±1.2	145	-8.8	.000*	-13.219	-8.35
	PV_3	1	1.6±0.1	18.4	13.8	-	1.32	1.793
		2	-.6±0.1	14.3	-3.8	.002*	-0.852	-0.238
		3	-.9±0.1	19.4	-6.9	.000*	-1.186	-0.633
		4	-1.0±0.1	23.9	.6.7	.000*	-1.261	-0.664
		5	-.9±0.2	44.3	-4.9	.000*	-1.305	-0.549
	u_{3t}	1	.3±0.03	145	9.3	-	0.206	0.316
		2	.1±0.04	145	2.7	.000*	0.026	0.165
		3	.002±0.03	145	0.05	0.958	-0.063	0.067
		4	.06±0.04	145	1.6	0.11	-0.013	0.13
		5	-.02±0.05	145	-0.4	0.711	-0.114	0.078
P2 MA	DS	2	52.2±3.8	9.9	13.6	-	43.672	60.744
		3	-15.7±4.9	11.9	-3.2	.008*	-26.379	-5.001
		4	-24.3±5.2	12.2	-4.7	.001*	-35.625	-13.009
		5	-21.0±4.5	11.1	-4.7	.001*	-30.796	-11.179
	STdur	2	74.7±2.6	10.8	28.4	-	68.919	80.526
		3	-8.7±3.3	12.6	-2.6	.022*	-15.989	-1.482
		4	-11.3±3.5	12.9	-3.2	.007*	-19.021	-3.678
		5	-11.9±3.1	11.9	-3.9	.002*	-18.575	-5.251
	PV_3	2	3.6±2.7	110	13.5	-	3.076	4.13
		3	-.6±0.4	110	-1.6	0.103	-1.331	0.125
		4	-2.0±0.4	110	-5.3	.000*	-2.706	-1.233
		5	-2.5±0.3	110	-7.9	.000*	-3.079	-1.84
	u_{3t}	2	.4±0.1	111	7.3	-	0.268	0.466
		3	-.006±0.07	111	-0.1	0.927	-0.141	0.129
		4	-.3±0.07	111	-4.4	.000*	-0.445	-0.168
		5	-.2±0.06	111	-2.7	.008*	-0.275	-0.043
P2 LA	DS	2	52.2±3.7	9.1	14	-	43.805	60.688
		3	-14.3±4.8	11.2	-3	.012*	-24.933	-3.755
		4	-25.5±5.1	11.5	-5	.000*	-36.655	-14.259
		5	-20.2±4.4	10.5	-4.6	.001*	-29.902	-10.456
	STdur	2	77.0±1.1	3	71	-	73.572	80.46
		3	-5.0±1.6	6.1	-3.2	.019*	-8.825	-1.134
		4	-13.4±1.7	6.4	-8	.000*	-17.509	-9.376
		5	-8.8±1.4	4.9	-6.3	.002*	-12.425	-5.251
	PV_2	2	18.3±1.1	5.1	16.6	-	15.525	21.161
		3	3.9±1.8	13.9	2.1	0.05	-0.008	7.863
		4	-2.7±1.7	9.2	-1.6	0.146	-6.538	1.135
		5	-3.4±1.4	7.3	-2.5	.039*	-6.655	-0.238
	PV_3	2	2.2±0.2	10.1	9.9	-	1.726	2.725
		3	0.2±0.3	17.5	-0.7	0.519	-0.927	0.485
		4	-.8±0.3	14.1	-2.5	.023*	-1.518	-0.131
		5	-1.0±0.3	12.2	-3.8	.003*	-1.585	-0.422
	u_{3t}	2	.2±0.1	112	3.7	-	0.102	0.341
		3	.01±0.1	112	0.1	0.906	-0.202	0.227
		4	.2±0.1	112	1.8	0.076	-0.019	0.369
		5	-.1±0.08	1123	-1.7	0.102	-0.281	0.026
P2 both	Velocity	2	.9±0.2	13.8	3.9	-	0.394	1.368
		3	.4±0.3	14.5	1.3	0.22	-0.241	0.9619

		4	1.2±0.3	15	3.8	.002*	0.514	1.79
		5	1.5±0.3	14.2	5.8	.000*	0.956	2.065
P3	Velocity	1	.6±0.05	12.161	11.8	-	0.498	0.724
		2	.01±0.07	13.598	0.2	0.834	-0.127	0.155
		3	.1±0.07	12.17	1.5	0.158	-0.049	0.27
		4	.2±0.06	13.131	3.8	.002*	0.1	0.37
		5	.05±0.08	14.159	0.7	0.501	-0.111	0.216
	DS	1	53.1±2.1	8.131	24.9	-	48.196	57.99
		2	-4.5±3.1	14.938	-1.5	0.166	-11.041	2.086
		3	-13.4±3.0	8.132	-4.4	.002*	-20.327	-6.472
		4	-16.5±2.8	13.081	-5.8	.000*	-22.611	-10.334
		5	-5.1±3.5	14.161	-1.5	0.161	-12.548	2.3
	STdur	1	78.5±1.3	6.209	62.5	-	75.424	81.522
		2	-1.9±1.8	10.882	-1.1	0.303	-5.874	2.005
		3	-7.2±1.8	6.211	-4.1	.006*	-11.521	-2.897
		4	-8.1±1.7	9.566	-4.9	.001*	-11.822	-4.396
		5	-2.4±2.0	10.293	-1.2	0.258	-6.887	2.058
	PV_2	1	17.6±1.7	112	10.4	-	14.238	20.968
		2	4.7±2.0	112	2.3	.022*	0.685	8.669
		3	4.5±2.0	112	2.3	.025*	0.571	8.373
		4	4.1±2.0	112	2.05	.043*	0.135	8.164
		5	10.4±2.0	112	4.7	.000*	6.039	14.782
	PV_3	1	2.3±0.4	112	6.6	-	1.625	3.025
		2	-.5±0.4	112	-1.2	0.248	-1.317	0.343
		3	-.7±0.4	112	-1.6	0.113	-1.466	0.157
		4	.6±0.4	112	1.4	0.158	-0.236	1.434
		5	3.4±0.5	112	7.4	.000*	2.493	4.311
	u_{3t}	1	.01±0.05	112	0.3	-	-0.083	0.107
		2	-.1±0.06	112	-1.8	0.081	-0.213	0.013
		3	-.02±0.6	112	-0.4	0.71	-0.131	0.09
		4	.08±0.06	112	1.3	0.187	-0.038	0.19
		5	-.01±0.06	112	-0.2	0.882	-0.133	0.114

Abbreviations: PV_2, percentage of total variation explained by the 2nd principal component; PV_3, percentage of total variation explained by the 3rd principal component; u_3t, orientation of the normal to the plane; DS, double support; STdur, stance duration; MA, most affected side; LA, least affected side; SD, standard deviation; CI, confidence interval.
