# Supplementary material for: Early Development of Locomotor Patterns and Motor Control in Very Young Children at High Risk of Cerebral Palsy, a Longitudinal Case Series
Source: Front Hum Neurosci. 2021 Jun 3;15:659415. doi: 10.3389/fnhum.2021.659415 (PMC8209291; doi:10.3389/fnhum.2021.659415)
Supplement: Supplementary file 2 [file Table_2.pdf]

Supplementary Table 2. LLM results FWHM muscle activity: estimates of fixed effects session

Participant	Parameter	Session	Estimate (mean±SD)	df	t	p-value	95% CI	
							Lower bound	Upper bound
P1	TA	1	8.5±1.3	222	6.7	-	6.013	11.033
		2	8.6±1.7	222	5	.000*	5.26	12.011
		3	6.8±1.6	222	4.2	.000*	3.608	9.983
		4	4.6±1.7	222	3.6	.009*	1.178	8.031
		5	13.0±1.9	222	6.8	.000*	9.221	16.711
	GM	1	5.7±1.2	222	4.9	-	3.365	7.947
		2	14.4±1.6	222	9.2	.000*	11.312	17.473
		3	15.6±1.5	222	10.6	.000*	12.699	18.518
		4	15.1±1.6	222	9.5	.000*	11.954	18.209
		5	17.9±1.7	222	10.3	.000*	14.518	21.354
	SOL	1	10.07±1.5	191	7.6	-	7.118	13.018
		2	5.2±2.0	191	2.6	.010*	1.271	9.206
		3	11.8±1.9	191	6.2	.000*	8.084	15.577
		4	11.7±2.0	191	5.7	.000*	7.648	15.703
		5	-	-	-	-	-	-
	RF	1	7.3±1.5	222	5	-	4.397	10.161
		2	9.9±2.0	222	5	.000*	6.035	13.788
		3	10.2±1.9	222	5.5	.000*	6.585	13.907
		4	5.6±2.0	222	2.8	.005*	1.664	9.534
		5	10.3±2.2	222	4.7	.000*	6.042	14.642
	VM	1	-	-	-	-	-	-
		2	24.1±1.3	184	17.9	-	21.43	26.734
		3	-7.6±1.8	184	-4.3	.000*	-11.1	-4.067
		4	-12.2±1.9	184	-6.3	.000*	-16.045	-8.418
		5	-10.1±2.1	184	-4.7	.000*	-14.261	-5.849
	VL	1	11.5±1.3	222	9.1	-	8.992	13.998
		2	-0.9±1.7	222	-0.05	0.599	-4.265	2.467
		3	7.6±1.6	222	4.7	.000*	4.464	10.822
		4	1.2±1.8	222	0.7	0.472	-2.169	4.665
		5	11.8±1.9	222	6.2	.000*	8.073	15.542
	BF	1	7.3±1.3	222	5.3	-	4.663	9.974
		2	12.2±1.8	222	6.7	.000*	8.588	15.729
		3	12.7±1.7	222	7.4	.000*	9.298	16.042
		4	6.4±1.8	222	3.5	.001*	2.795	10.044
		5	22.6±2.0	222	11.3	.000*	18.683	26.606
	TFL	1	-	-	-	-	-	-
		2	20.6±1.5	153	14	-	17.72	23.521
		3	-6.1±1.9	153	-3.1	.002*	-9.982	-2.291
		4	-3.6±2.1	153	-1.7	0.087	-7.808	0.534
		5	-	-	-	-	-	-
	GLM	1	-	-	-	-	-	-
		2	14.4±1.4	184	10.4	-	11.697	17.18
		3	2.9±1.8	1884	1.6	0.117	-0.733	6.537
		4	5.7±2.0	184	2.9	.005*	1.757	9.642
		5	8.3±2.2	184	3.8	.000*	3.922	12.619
	ES	1	11.1±1.8	221	6.2	-	7.567	14.646
		2	13.9±2.4	221	5.8	.000*	9.133	18.596
		3	12.4±2.3	221	5.4	.000*	7.892	16.837
		4	7.8±2.4	221	3.2	.002*	2.986	12.59
		5	13.2±2.7	221	5	.000*	7.998	18.481
P2 MA	TA	2	13.6±1.0	173	13.9	-	11.662	15.522
		3	-2.5±1.6	173	-1.6	0.119	-5.712	0.659
		4	5.6±1.5	173	3.8	.000*	2.68	8.47
		5	3.4±1.4	173	2.5	.013*	0.746	6.103
	GM	2	11.6±1.7	170	7	-	8.344	14.923
		3	-2.3±2.7	170	-0.8	0.402	-7.592	3.059
		4	11.0±2.5	170	4.5	.000*	6.139	15.843
		5	16.0±2.3	170	7	.000*	11.452	20.45
	GL	2	-	-	-	-	-	-
		3	11.0±1.9	123	5.8	-	7.24	14.822
		4	7.1±2.5	123	2.8	.006*	2.074	12.033
		5	7.2±2.4	123	3	.003*	2.455	11.856
	SOL	2	14.2±1.0	171	14	-	12.18	16.178
		3	-6.4±1.7	171	-3.7	.000*	-9.742	-2.991
		4	-3.1±1.5	171	-2	.043*	-6.095	-0.099
		5	-2.3±1.4	171	-1.6	0.107	-5.049	0.498
	RF	2	10.8±1.4	175	7.9	-	3.134	13.545
		3	2.0±2.3	175	0.9	0.394	-2.563	6.479

		4	12.3±2.1	175	5.9	.000*	8.241	16.447
		5	7.2±1.9	175	3.8	.000*	3.418	11
	VM	2	7.2±0.8	173	8.5	-	5.547	8.881
		3	4.8±1.4	173	3.4	.001*	2.06	7.564
		4	9.0±1.3	173	7.1	.000*	6.492	11.493
		5	10.3±1.2	173	8.8	.000*	7.95	12.577
	VL	2	8.6±1.1	164	8.2	-	6.525	10.695
		3	5.6±1.7	164	3.2	.002*	2.129	9.045
		4	10.9±1.6	164	6.9	.000*	7.794	14.015
		5	7.4±1.5	164	4.9	.000*	4.404	10.365
	BF	2	11.6±1.1	167	10.1	-	9.297	13.819
		3	-2.6±2.1	167	-1.2	0.22	-6.841	1.589
		4	8.4±1.7	167	4.8	.000*	4.926	11.784
		5	13.7±1.6	167	8.5	.000*	10.51	16.845
	TFL	2	9.8±1.1	171	8.8	-	7.617	12.055
		3	1.9±1.8	171	1.1	0.289	-1.666	5.565
		4	6.9±1.7	171	4.1	.000*	3.601	10.184
		5	3.7±1.5	171	2.4	.019*	0.621	6.72
	GLM	2	8.0±1.0	170	9	-	6.935	10.806
		3	-2.0±1.6	170	-1.2	0.219	-5.091	1.177
		4	3.4±1.4	170	2.3	.022*	0.501	6.211
		5	6.1±1.3	170	4.5	.000*	3.45	8.794
	ES	2	10.2±1.1	175	9.1	-	7.966	12.393
		3	6.6±1.9	175	3.5	.001*	2.882	10.28
		4	7.8±1.7	175	4.6	.000*	4.411	11.125
		5	6.9±1.6	175	4.4	.000*	3.837	10.039
**P2 LA**	GM	2	14.8±1.5	171	10.1	-	11.896	17.896
		3	4.6±2.5	171	1.9	0.066	-0.305	9.602
		4	8.2±2.2	171	3.7	.000*	3.777	12.56
		5	9.2±2.1	171	4.4	.000*	5.061	13.251
	SOL	2	11.8±1.4	173	8.4	-	8.997	14.539
		3	4.5±2.3	173	1.9	0.059	-0.17	9.092
		4	3.5±2.1	173	1.7	0.099	-0.672	7.733
		5	9.0±2.0	173	4.5	.000*	5.113	12.95
	RF	2	13.1±1.2	173	11.3	-	10.843	15.392
		3	4.0±1.9	173	2.1	.041*	0.169	7.771
		4	4.4±1.7	173	2.5	.012*	0.971	7.87
		5	5.3±1.6	173	3.3	.001*	2.112	8.546
	VM	2	11.4±1.3	173	9	-	8.897	13.893
		3	-.5±2.1	173	-0.3	0.8	-4.71	3.639
		4	9.4±1.9	173	4.9	.000*	5.588	13.165
		5	7.3±1.8	173	4.1	.000*	3.739	10.804
	VL	2	15.1±1.5	173	9.9	-	12.14	18.151
		3	3.5±2.5	173	1.4	0.17	-1.52	8.525
		4	6.5±2.3	173	2.8	.005*	1.939	11.054
		5	.3±2.1	173	0.2	0.873	-3.906	4.595
	BF	2	10.1±1.2	168	8.4	-	7.746	12.518
		3	1.8±2.1	168	0.9	0.39	-2.359	6.015
		4	10.2±1.8	168	5.5	.000*	6.545	13.834
		5	8.7±1.7	168	5.1	.000*	5.352	12.1
	TFL	2	9.5±1.2	173	8.1	-	7.189	11.831
		3	5.0±2.0	173	2.5	.012*	1.107	8.865
		4	7.9±1.8	173	4.4	.000*	4.42	11.46
		5	6.6±1.7	173	3.9	.000*	3.271	9.836
	GLM	2	9.4±1.1	172	8.9	-	7.278	11.436
		3	2.7±1.8	172	1.5	0.128	-0.789	6.24
		4	9.3±1.6	172	5.8	.000*	6.171	12.478
		5	3.5±1.5	172	2.3	.022*	0.513	6.394
	ES	2	12.0±1.2	173	9.7	-	9.556	14.439
		3	4.1±2.1	173	2	.048*	0.031	8.191
		4	9.0±1.9	173	4.8	.000*	5.32	12.725
		5	7.8±1.7	173	4.4	.000*	4.326	11.231
**P3**	TA	1	7.8±.8	254	9.9	-	6.22	9.302
		2	2.6±1.3	254	2	.046*	0.048	5.216
		3	3.5±1.1	254	3.2	.002*	1.345	5.703
		4	4.1±1.2	254	3.5	.000*	1.84	6.437
		5	4.2±1.2	254	3.6	.000*	1.873	6.445
	GM	1	6.2±1.4	218	4.3	-	3.345	9
		2	-	-	-	-	-	-
		3	1.1±2.0	218	0.5	0.587	-2.883	5.082
		4	15.4±2.1	218	7.2	.000*	11.215	19.612
		5	9.6±2.1	218	4.5	.000*	5.423	13.774
	GL	1	15.8±1.9	254	8.3	-	12.042	19.534

	2	6.5±3.1	254	2.1	.038*	0.372	12.715
	3	4.6±2.7	254	1.7	0.086	-0.654	9.941
	4	3.0±2.8	254	1	0.287	-2.561	8.614
	5	8.8±2.8	254	3.1	.002*	3.223	14.336
SOL	1	14.6±1.6	254	9	-	11.396	17.825
	2	6.3±2.7	254	2.3	.020*	1.004	11.595
	3	-7.3±2.3	254	-3.2	.002*	-11.851	-2.759
	4	6.0±2.4	254	2.5	.015*	1.187	10.775
	5	6.4±2.4	254	2.7	.008*	1.667	11.202
VM	1	10.3±1.3	95	8.1	-	7.8	12.847
	2	7.0±2.1	95	3.4	.001*	2.889	11.204
	3	-	-	-	-	-	-
	4	-	-	-	-	-	-
	5	-	-	-	-	-	-
VL	1	17.6±1.5	254	12	-	14.725	20.532
	2	5.7±2.4	254	2.4	.019*	0.957	10.524
	3	4.9±2.1	254	2.3	.020*	0.778	8.99
	4	1.5±2.2	254	0.7	0.496	-2.83	5.831
	5	-.2±2.1	254	-0.09	0.925	-4.512	4.101
BF	1	10.3±1.3	252	7.9	-	7.738	12.908
	2	9.3±2.2	252	4.3	.000*	5.077	13.594
	3	8.1±1.9	252	4.4	.000*	4.42	11.732
	4	6.3±2.0	252	3.2	.002*	2.412	10.123
	5	6.0±2.0	252	3	.003*	2.124	9.879
TFL	1	6.2±1.2	254	5.2	-	3.819	8.502
	2	11.2±2.0	254	5.7	.000*	7.337	15.053
	3	11.9±1.7	254	7.1	.000*	8.62	15.244
	4	9.7±1.8	254	5.5	.000*	6.206	13.191
	5	9.2±2.8	254	5.2	.000*	5.749	12.695
GLM	1	-	-	-	-	-	-
	2	10.8±1.1	194	9.7	-	8.601	12.949
	3	-2.7±1.4	194	-1.9	0.055	-5.409	0.062
	4	4.7±1.4	194	3.2	.001*	1.842	7.535
	5	4.3±1.4	194	3	.003*	1.505	7.174
ES	1	-	-	-	-	-	-
	2	10.1±1.2	194	8.7	-	7.854	12.444
	3	-1.5±1.5	194	-1	0.323	-4.339	1.437
	4	.9±1.5	194	0.6	0.534	-2.056	3.953
	5	2.7±1.5	194	1.8	0.076	-0.286	5.699

Abbreviations: TA, tibialis anterior; GM, gastrocnemius medialis; GL, gastrocnemius lateralis; SOL, soleus; RF, rectus femoris; VM, vastus medialis; VL, vastus lateralis; BF, biceps femoris; TFL, tensor fascia latae; GLM, gluteus maximus; ES, erector spinae at L2 level; MA, most affected side; LA, least affected side; SD, standard deviation; CI, confidence interval.
