# Supplementary material for: Early Development of Locomotor Patterns and Motor Control in Very Young Children at High Risk of Cerebral Palsy, a Longitudinal Case Series
Source: Front Hum Neurosci. 2021 Jun 3;15:659415. doi: 10.3389/fnhum.2021.659415 (PMC8209291; doi:10.3389/fnhum.2021.659415)
Supplement: Supplementary file 3 [file Table_3.pdf]

Supplementary Table 3. The percentage of strides with coefficients of correlation (r) higher than the predefined threshold (>0.3) between raw EMG signals (30 Hz cut-off high-pass) of indicated pairs of muscles. The mean value of r (found over the samples r>03 of data) is reported in brackets.

	TA-SOL	TA-GM	RF-BF	RF-TFL	GLM-TFL
Right Leg	0.9% (0.36)	0%	7.3% (0.49)	0.9% (0.41)	9.9% (0.49)
Left Leg	7.0% (0.47)	0.1% (0.31)	9.2% (0.43)	0.1% (0.30)	5.5% (0.43)
