# Supplementary material for: Early Development of Locomotor Patterns and Motor Control in Very Young Children at High Risk of Cerebral Palsy, a Longitudinal Case Series
Source: Front Hum Neurosci. 2021 Jun 3;15:659415. doi: 10.3389/fnhum.2021.659415 (PMC8209291; doi:10.3389/fnhum.2021.659415)

Supplementary Material 1

Full-Width of the Half Maximum (FWHM) of the mean muscle activity for each muscle, and session in P1-P3. TA, tibialis anterior; GM, gastrocnemius medialis; GL, gastrocnemius lateralis; SOL, soleus; RF, rectus femoris; VM, vastus medialis; VL, vastus lateralis; BF, biceps femoris; TFL, tensor fascia latae; GLM, gluteus maximus; ES, erector spinae at L2 level.

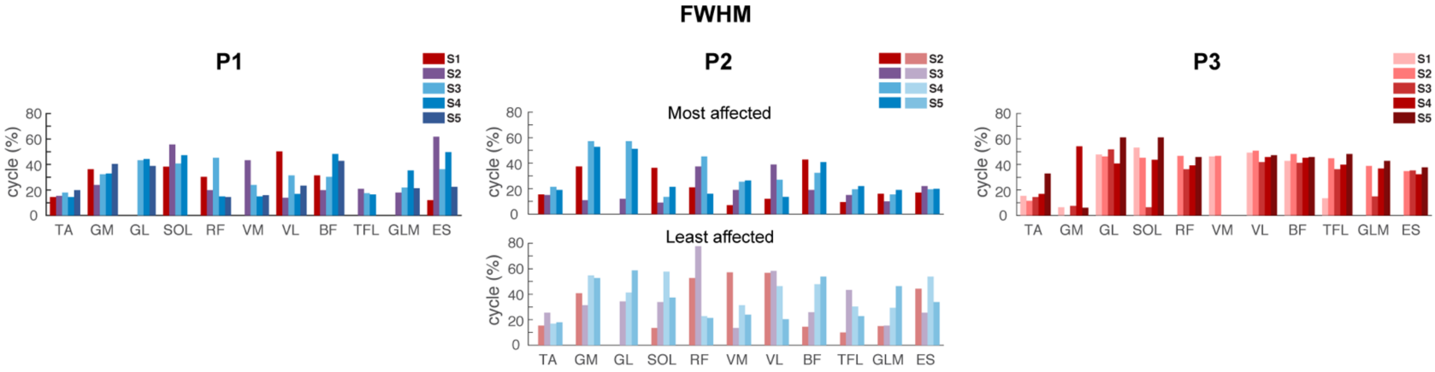

Supplement: Supplementary file 4 [file Data_Sheet_1.pdf]
